# On the Use of Creosote in Phthisis

**Published:** 1891-08-22

**Authors:** 


### THERAPEUTICS.

### ON THE USE OF CREOSOTE IN PHTHISIS.

A hundred and fifty years ago, Berkeley, the author of "The Theory of Vision," one of the greatest of English philosophers, astonished the world by an elaborate treatise on the virtues of tar water. His work was a master-piece in literature, and his eloquence gave fame for a time to a substance which had some real merits of its own, but the medicine failed to answer the great expectations which were raised of it and gradually fell into nearly complete disuse. When we reflect that tar is only a crude form of the much used carbolic acid, we see how useless are the most powerful drugs where there exists but scanty physical science to direct their use. Antiseptics might have been then introduced had there been the science to analyse the effects of tar water, but Berkeley, like a true poet, could only divine dimly that which patient study would at last render available for practical use.

In 1833, or rather earlier, Reichenbach discovered a product of the distillation of wood-tar, which he termed creosote, from its power of preserving flesh from putrefaction. It was quickly applied to various uses, and Keats introduced it as a therapeutic agent. Like many new remedies it found numerous friends who took it up for a time. Seven treatises by different authors appeared in 1833 discussing its merits, and in 1834 E. Miquet and Cormack gave complete accounts of all that was then known of it. Elliotson tried it in various diseases, and administered it by inhalation for phthisis. With the exception of its action in preventing flatulence, its virtues also proved disappointing upon longer trial. Yet in dentistry, and the arts other than that of therapeutics, it found many applications. Of late years it has even in these been largely superseded by the closely related product of coal tar, carbolic acid. From this it differs notably in not coagulating albumen, and probably in other less known but equally important effects on the animal organism. Ordinary creosote is not only a mixture of cresol, methyl-cresol, phlorol, and guiacol, but it contains a number of impurities, and very frequently that which is sold in the shops is nothing but carbolic acid of bad quality, and has not been made from wood-tar at all.

As its composition and impurities became known, i almost ceased to be employed in medicine until abou eleven years ago, when Gimbert and Bouchard revived its use, giving it by inhalation and otherwise. Sommerbrodt then administered it during more than nine years to some 5,000 patients, and came to the conclusion that it had enormous value, especially in the earlier stages of phthisis. He gave it in wine, in cod liver oil, and particularly in Reuss' capsules, each containing $\frac{1}{4}$ of a grain with three minims of balsam of Tolu. Commencing with one capsule, he increased the dose to six three times a-day, which he continued for months together. The effects on the appetite and general health were remarkable, and Sommerbrodt insists, in addition, that it actively hinders the multiplication of the bacillus. P. Guttman showed that the bacilli failed to develop in a medium containing a little over 1 in 4,000 of creosote, and others declare that even 1 in 12,500 has the same effect on them. Bouchard again has laid down that it requires fifteen grammes given during 24 hours to form a fatal dose for a man of 60 kilogrammes in weight. Can we then administer such a quantity continuously as will prevent the development of bacilli in the fluids of the body without producing poisonous effects on the patient? Bouchard thinks that, as three grammes daily is never necessary, it should be possible to destroy the activity of the germs without injurious results. Guttman holds that it would not be feasible to give a sufficiently large dose, and that we cannot tell how much of what is taken, actually circulates in the blood. Sommerbrodt taking the opposite view, and giving his patients about eleven grains daily, when they are fully accustomed to the drug, supposes that an accumulation in the blood takes place, so that the necessary amount is reached. Certainly his success with the drug is in favour of his theory, but there is so far no complete evidence of the truth of his view as to its action. Practically it is found that the greatest success is obtained when patients take the maximum dose which does not cause inconvenience, and continue its use for long periods.

Gimbert gives it often by injection, and Slackiewitz introduces these into the lung itself. Others employ inhalation or administer it by the rectum. By Rosenbusch a solution of a fifth per cent. in almond oil was employed for intrapulmonary injections. He inserts the needle in the second inter-costal space or in the supraspinous fossæ, but these injections do not appear to be free from risk, and as there are few patients who cannot take creosote in pills, capsules, or solutions, there is hardly reason for employing them. Mignon, however, states that the local antiseptic action is thus obtained most perfectly, and is of higher importance than any supposed direct action on the growth of bacilli. The great majority of observers have contented themselves, notwithstanding, with other methods of administration. An emulsion with cod-liver oil has found some favour, but has the disadvantage of combining two disagreeable flavours and of often creating a distaste for the oil itself. Schetelig injected 16 drops of a 25 per cent. solution with almond oil under the skin of the abdomen, and repeats the injection several times until even 45 minims of creosote are introduced in a day. In other cases he used guiacol in doses of $\frac{1}{3}$ or $\frac{1}{4}$ of those of creosote. Guiacol, though more expensive, is of a definite composition and has been preferred by many authorities among whom are Sahli, Fräntzel, and Junet. The latter declares that creosote has done more for phthisis than any other drug. Hopman again has tried creosote in several thousands of cases giving nine grains and upwards daily in tincture of gentian and found great general improvement with increase of appetite and weight. One most remarkable case is that of Dr. Bogdanovitch himself, who, finding no benefit from small doses, increased them, till he was taking 44 grains daily with extraordinary success. His fever, cough, and laryngeal spasm left him, and his general health became once more vigorous. However, the bacilli by no means disappeared from his sputa. The only inconvenience he felt was occasional giddiness and palpitation when the drug was taken on an empty stomach. Children were found by Sollman to bear the drug very well in doses of two to seven drops.

Among the various methods of administering it which have been employed, probably none are better than a solution containing a drachm each of creosote, oil of almonds, and spirits of chloroform, with rectified spirits to an ounce. A dose of ten minims gradually increased is taken in milk, wine, or water, by most patients surprisingly well. It has also been given with glycerine and whisky, or simply in milk, and in aërated water with a little spirit. When the solution caused nausea we have given it in coated pilules, but damp and uncoated ones are more disagreeable than solutions.

Occasionally diarrhœa appears to follow its use, and a smaller dose or a few days' rest is necessary, otherwise the great rule seems to be to keep up constantly the administration for months together. If care is not taken to obtain a pure quality we may easily get carbolic acid poisoning, and probably to this circumstance the recorded instances of nephritis and anasarca are due. It is said to be contra-indicated where there is a tendency to hæmoptysis, or in very advanced cases, or with lardaceous disease, or in ulceration of the bowels, though in the latter it may possibly succeed in arresting the local mischief. Robert Hare found that animals poisoned by very large doses were saved by the administration of the soluble sulphates, such as Epsom salts, which form with it harmless sulpho-carbolates.

On looking over the records of the many observers who have employed it, we cannot doubt that we have in creosote a most valuable remedy in the milder forms of phthisis. Whatever is its real mode of action, whether it has a direct action on the bacilli, or whether it prevents putrid intoxication, suitable patients when using it rapidly gain weight and strength, and lose more or less of the cough and expectoration. In the mildest cases a complete cure may follow. The mode of administration may be changed if nausea and repugnance arise, but it seems important to keep up the ingestion of some ten or fifteen minims daily for a considerable period, and to supplement it with the most careful attention to hygiene and feeding.

